# IL-13/STAT6 signaling plays a critical role in the epithelial-mesenchymal transition of colorectal cancer cells

**DOI:** 10.18632/oncotarget.11282

**Published:** 2016-08-13

**Authors:** Hui Cao, Jing Zhang, Hong Liu, Ledong Wan, Honghe Zhang, Qiong Huang, Enping Xu, Maode Lai

**Affiliations:** ^1^ Department of Pathology, School of Medicine, Zhejiang University, Hangzhou 310058, China; ^2^ Key Laboratory of Disease Proteomics of Zhejiang Province, Hangzhou 310058, China; ^3^ Department of Pathology and Pathophysiology, Cheng Du Medical College, Chengdu 610500, China; ^4^ Jinhua People's Hospital, Jinhua Polytechnic College, Jinhua 321000, China

**Keywords:** colorectal cancer, interleukin-13, STAT6, ZEB1, EMT

## Abstract

Colorectal cancer (CRC) is one of the most common causes of cancer-related death worldwide due to the distant metastases. Compelling evidence has reported that epithelial-mesenchymal transition (EMT) is involved in promoting cancer invasion and metastasis. However, the precise molecular events that initiate this complex EMT process remain poorly understood. Here, we showed that the pleiotropic cytokine interleukin-13 (IL-13) could induce an aggressive phenotype displaying EMT by enhancing the expression of EMT-promoting factor ZEB1. Importantly, STAT6 signaling inhibitor and STAT6 knockdown significantly reversed IL-13-induced EMT and ZEB1 induction in CRC cells, whereas ectopic STAT6 expression in STAT6^null^ CRC cell line markedly promoted EMT in the present of IL-13. ChIP-PCR and Luciferase assays revealed that activated STAT6 directly bound to the promoter of *ZEB1*. Otherwise, we found IL-13 also up-regulated the stem cell markers (nanog, CD44, CD133 and CD166) and promoted cell migration and invasion through STAT6 pathway. We also found that siRNA-mediated knockdown of IL-13Rα1 could reverse IL-13-induced ZEB1 and EMT changes by preventing STAT6 signaling. Finally, we demonstrated positive correlation between IL-13Rα1 and ZEB1 at mRNA levels in human CRC samples. Taken together, our findings first demonstrated that IL-13/IL-13Rα1/STAT6/ZEB1 pathway plays a critical role in promoting EMT and aggressiveness of CRC.

## INTRODUCTION

Colorectal cancer (CRC) is one of the most fatal neoplastic diseases worldwide. Although improved treatment strategies have increased the overall survival rates in the early stages, 40–50% of all CRC patients present with metastasis either at the diagnosis or as recurrent disease upon intended curative therapy [1]. Most CRC patients with distant metastasis are not suitable candidates for conventional intervention and exhibit a poor five-year survival rate of < 10% [2]. From a therapeutic perspective, defining the molecular mechanisms underlying metastatic progression of CRC may contribute to decreasing morbidity and mortality [3].

The mutual and interdependent interactions between cancer cells and their microenvironment are important determinants of cancer progression toward metastasis [4]. Interleukins (IL) comprises a superfamily of pleiotropically acting cytokines that are present in the tumor microenvironment [5]. IL-13, a key T helper 2 (Th2) cell-derived cytokine, has been shown to be involved in regulating normal physiological processes including inflammation, immune response, mucus production and tissue reconstruction, but also in some pathological process such as autoimmune diseases, bronchial asthma and organ fibrosis [6, 7]. In particular, IL-13 has a central role in the pathogenesis of ulcerative colitis (UC) [8], a major type of inflammatory bowel disease (IBD) that has a significantly increased risk of CRC [9]. Elevated levels of IL-13 were detected in some tumors, such as breast cancer [10], oral squamous cell carcinoma [11] and colorectal cancer [12]. IL-13 signaling is initiated by binding to the IL-13 receptor alpha 1 (IL-13Rα1), which forms a receptor complex with IL-4 receptor α chain (IL-4Rα) and mediates signal transduction through the canonical JAK/STAT6 pathway [13]. In Hodgkin's lymphoma (HL), IL-13 promotes proliferation and inhibits cells death through STAT6 activation [14–16]. STAT6 is a transcription factor which can be activated by phosphorylation in the presence of some cytokines and growth factors such as IL-4 and IL-13. Phosphorylated STAT6 translocates to the nucleus and binds to regulatory promoter elements with the core sequence motif 5′-TTC(N)2-4GAA-3′ [17, 18]. Previous studies have shown that STAT6 could directly control the transcription of various genes (such as *Bcl-xL* [16], *LRH-1* [19], *LMP1* [20], *ORMDL3* [21]) in response to IL- 13. Recent years, IL-13Rα2, a so-called decoy receptor [22, 23], has been shown to be highly expressed in many tumor types, such as head and neck, glioblastoma and lung [24–26], and was also shown to promote invasion and metastasis of colorectal, ovarian and pancreatic cancers [27–29]. Intriguingly, IL-13 has also been reported to activate tumor-associated macrophages (TAMs), which promotes proliferation, survival and metastasis of tumor cells [30]. Thus, the underlying mechanism of IL-13 contributing to CRC progression needs to be further explored.

It is widely accepted that the developmental program termed epithelial-mesenchymal transition (EMT) plays a critical role in promoting carcinoma invasion and metastasis. The EMT program allows the epithelial cells to disrupt cell-cell adherence, lose apical-basal polarity, dramatically remodel the cytoskeleton and finally acquire mesenchymal phenotypes such as enhanced migratory capacity and invasiveness [31]. TGF-β and IL-13 have been shown to play a synergistic role in the pathogenesis of intestinal fistulae by inducing EMT program [32]. However, the function and mechanism of IL-13 in cancer EMT and aggressiveness are still unknown now. In the present study, we first found the role of IL-13 in promoting EMT and enhancing aggressiveness of CRC cells. Our study provides further insight into the exploring of IL-13/IL-13Rα1/STAT6/ZEB1 signaling as a novel target in potential CRC therapy.

## RESULTS

### IL-13 induces EMT phenotypes in CRC cells

**Figure 1:**
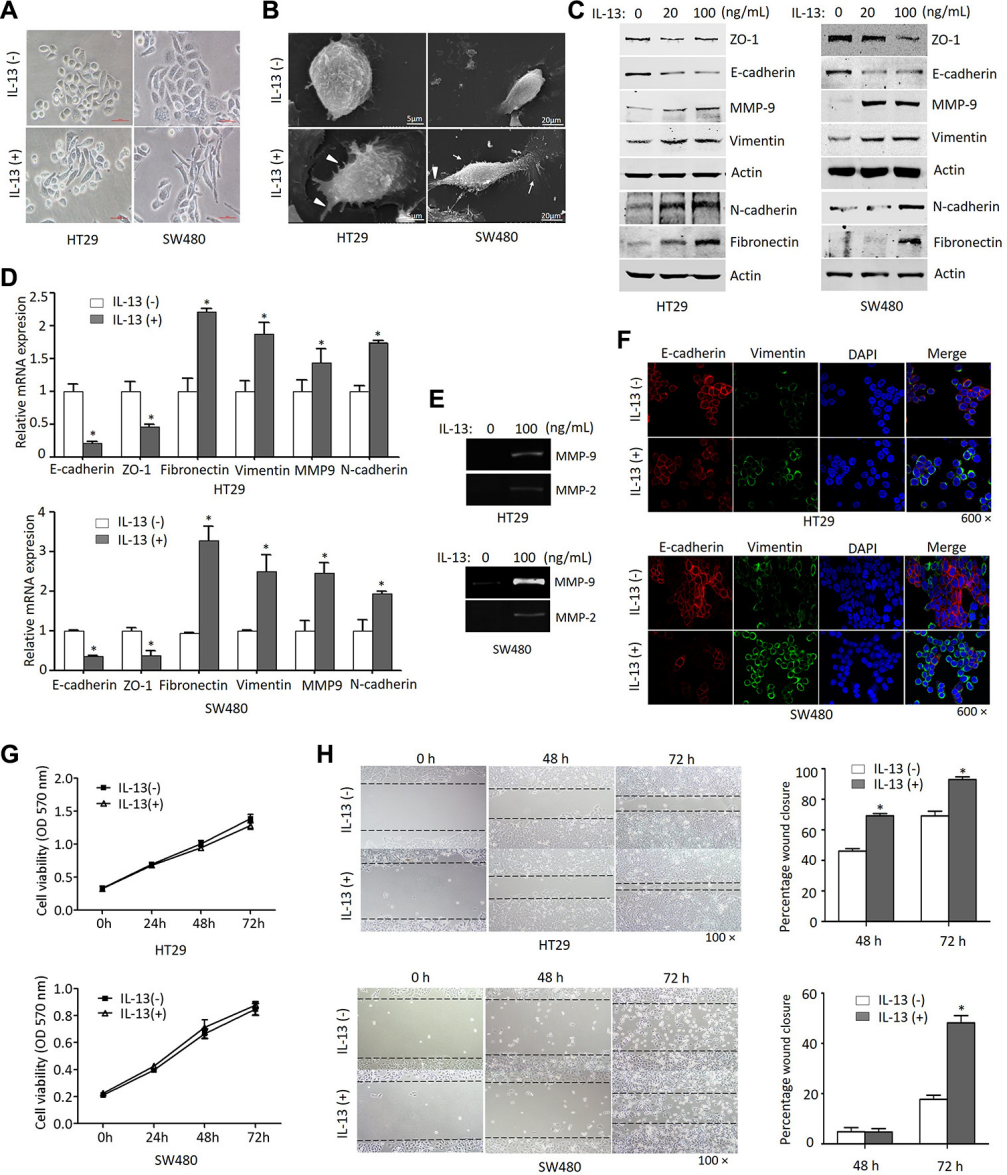
IL-13 induces an EMT phenotype in CRC cells (**A**) Morphology of HT29 and SW480 cells treated with or without IL-13 (100 ng/mL) for 72 h under phase contrast microscopy. Scale bar = 100 μm. (**B**) Cells treated with IL-13 (100 ng/mL) showed increased microvillin (*White triangle*) and pseudopodium (*white arrows*) under scanning electron microscopy, Scale bar = 5 μm or 20 μm. (**C**) Immunoblot analysis for EMT markers from 20 ng/mL or 100 ng/mL IL-13-treated cells and control cells. (**D**) Transcription levels of EMT markers determined by qRT-PCR (normalized to GAPDH). *P < 0.05. (**E**) Gelatin zymography for MMPs activity in conditioned medium of 100 ng/mL IL-13-treated HT29 and SW480 cells. (**F**) Immunofluorescent staining of E-cadherin (red) and Vimentin (green) expression in 100 ng/mL IL-13-induced HT29 and SW480 cells (nuclei stained with DAPI, 600×). (**G**) CCK8 analysis of the proliferation of HT29 and SW480 cells treated with IL-13 (100 ng/mL). (**H**) The migration of HT29 and SW480 cells stimulated by 100 ng/mL IL-13 for 0 h, 48 h and 72 h was detected by wound-healing assay (100×). Relative wound width represented as percentages compared with the wound width at 0 h. Error bars represent SD. *P < 0.05.

Elevated levels of IL-13 have been shown in colorectal cancer (CRC) [12], we set out to determine the potential role of IL-13 in EMT induction in CRC cells. After being exposed to IL-13 for 72 h, the morphological changes of HT29 and SW480 cells were observed. Under the optical microscope, the cells displayed cobblestone-like phenotypes and formed islets in the absence of IL-13. However, in the presence of IL-13 both groups of cells acquired a more fibroblast-like, spindle-shaped morphology indicative of mesenchymal cells (Figure 1A). Under scanning electron microscope, IL-13-treated cells showed increased microvilli and pseudopodium (Figure 1B). The morphological transformation indicated that cells incubated with IL-13 might undergo EMT-related changes. As expected, IL-13 treatment of HT29 and SW480 cells markedly decreased epithelial markers E-cadherin and ZO-1 expression and increased the expression of mesenchymal markers Vimentin, MMP9, N-cadherin and Fibronectin, as analyzed by immunoblotting and qRT-PCR assays (Figure 1C and 1D). Furthermore, the increased MMP activities were verified by gelatin zymography (Figure 1E). Similarly, immunofluorescence assay also showed that E-cadherin was significantly inhibited and Vimentin was obviously induced by IL-13 in HT29 and SW480 cell lines (Figure 1F). In addition, we found IL- 13 had no effect on the proliferation status of HT29 and SW480 cells by using CCK8 assay (Figure 1G). To determine the effect of IL-13 on the migration of CRC cells, wound-healing assay was performed in HT29 and SW480 cells. The results showed that the area changes for wound healing were enhanced in the present of IL-13 (*P* < 0.05) (Figure 1H). Taken together, these data demonstrated that IL-13 exposure leads to EMT process and migration in CRC cells.

### ZEB1 is a major player in the IL-13 induced EMT

**Figure 2:**
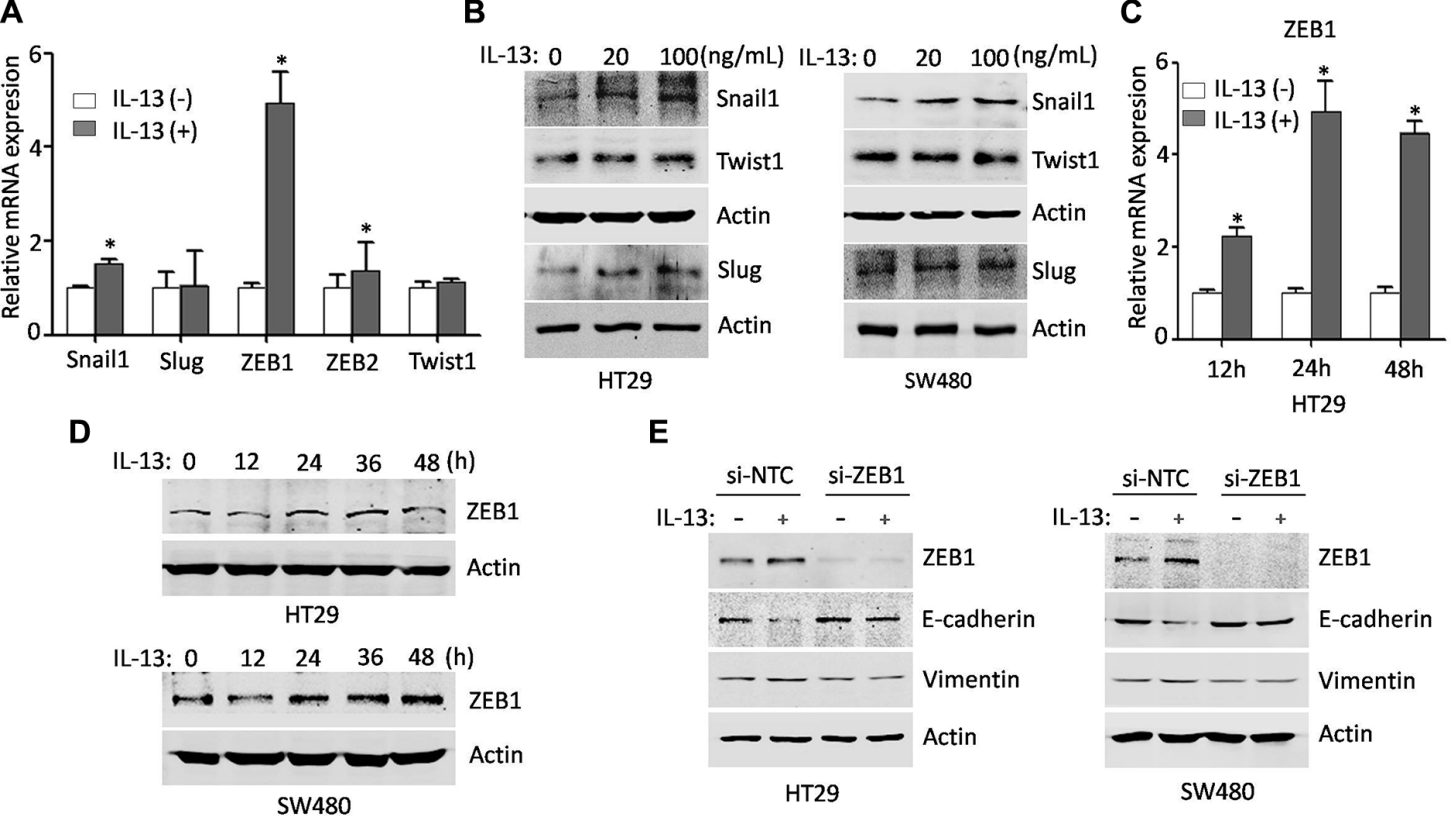
ZEB1 is a major player in the IL-13 induced EMT (**A**) Real-time PCR analysis of EMT core regulators in HT29 cells treated with or without IL-13 (100 ng/mL) for 24 h (normalized to GAPDH). Error bars represent SD. *P < 0.05. (**B**) Protein levels of EMT-related transcriptional factors in control and IL-13-treated HT29 and SW480 cells. (**C**) qRT-PCR analysis of transcription levels of ZEB1 in HT29 cells treated with or without IL-13 (100 ng/mL) for different time points. GAPDH was used as an internal normalization control. Error bars represent SD. *P < 0.05. (**D**) Immunoblot analysis for ZEB1 from HT29 and SW480 cells treated with 100 ng/mL IL-13 for indicated times. (**E**) Western blots of ZEB1, Vimentin and E-cadherin from HT29 and SW480 cells transfected with control or ZEB1 siRNA and incubated with IL-13 for 72 h.

The EMT process is initially driven by a set of key transcription factors including Snail1, Slug, ZEB1, ZEB2, Twist1 and Twist2. These EMT core regulators are able to suppress E-cadherin directly or indirectly, which is a gatekeeper of the epithelial state in carcinoma [33]. To identify the factors that mediated IL-13-induced EMT in CRC cells, we first examined the expression patterns of EMT core regulators by qRT-PCR (Figure 2A). It was found that the mRNA levels of ZEB1, ZEB2 and Snail1, but not Slug and Twist1, were up-regulated in IL-13-induced HT29 cells when compared with untreated cells, and the change of ZEB1 was the most obvious. The changes of protein levels were also verified (Figure 2B). Next, we further examined the ZEB1 expression at mRNA and protein levels in HT29 and SW480 cells by treating these cells with IL-13 for different time points (Figure 2C and 2D). The results showed that IL-13 resulted in a robust increase in ZEB1 expression. Next, we aimed to further investigate the important role of ZEB1 in the IL-13-induced EMT process. When ZEB1-siRNA was transferred into HT29 and SW480 cells, the IL-13-induced expression changes in EMT markers (a decrease of E-cadherin and induction of Vimentin) were blunted in the ZEB1-silenced CRC cells (Figure 2E). These results revealed that ZEB1 tansactivation is essential for IL-13-induced EMT program in CRC cells.

### IL-13 promotes EMT changes via the phosphorylation of STAT6 in CRC cells

**Figure 3:**
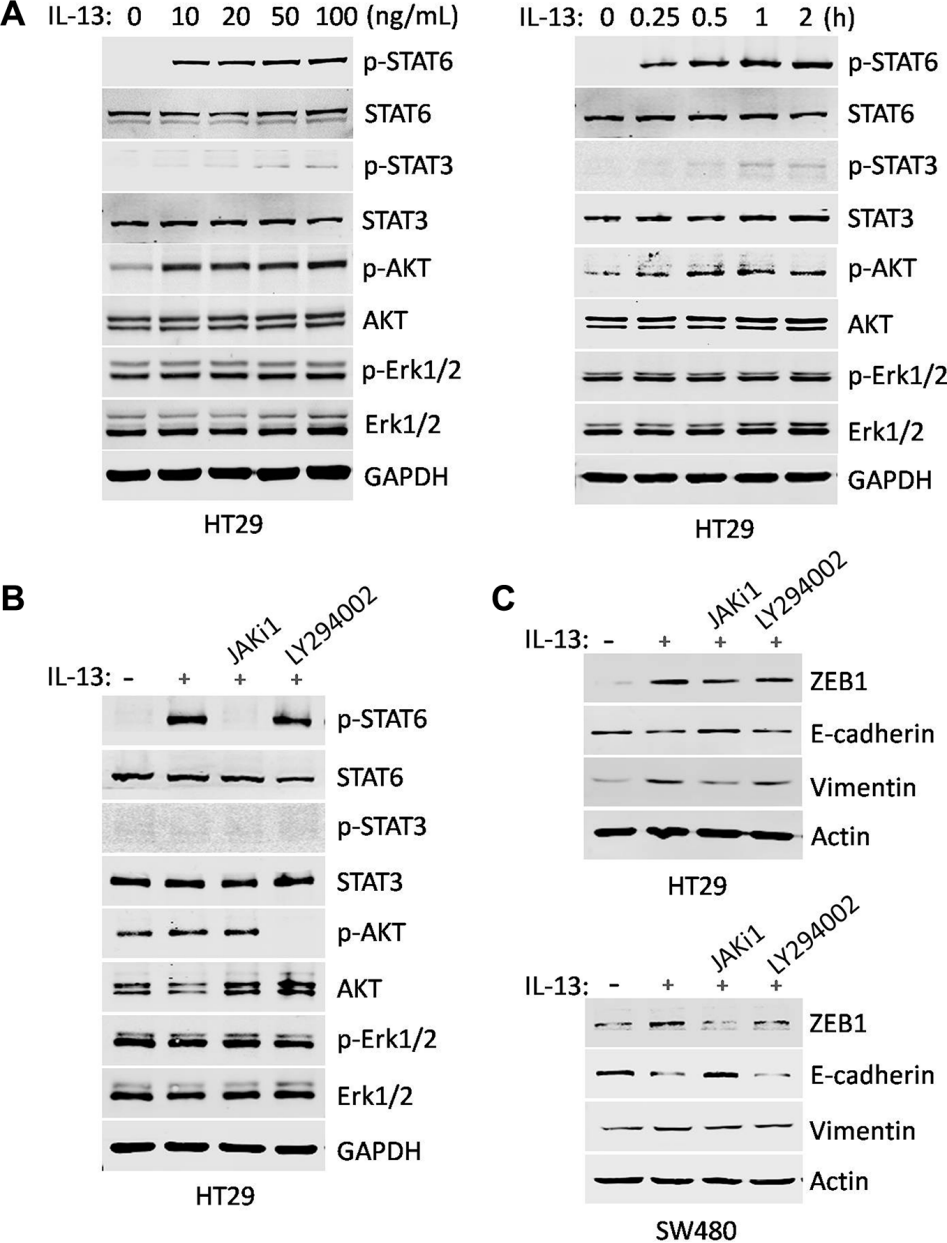
IL-13-induced EMT changes are mediated by JAK/STAT6 activation (**A**) HT29 cells were treated with gradient concentrations of IL-13 for 1 h (Left) and 100 ng/mL IL-13 for different time points (Right). The phosphorylation levels of STAT6, STAT3, AKT and Erk1/2 were examined by immunoblotting. STAT6, STAT3, AKT, Erk1/2 and GAPDH were used as sample loading controls. (**B**) Immunoblot analysis for the inhibition effect of signaling inhibitors in HT29 cells treated with JAK/STAT6 signaling inhibitor JAKi1 (10 uM) and PI3K signaling inhibitor LY294002 (0.1 uM) for 1 h. GAPDH were used as sample loading controls. (**C**) Expression of ZEB1, E-cadherin and Vimentin was examined by immunoblotting in HT29 and SW480 cells pretreated for 1 h with 10 μM JAKi1 or 0.1 μM LY294002 and exposed to 100 ng/mL IL-13 for additional 72 h. Actin was used as a loading control.

We found that IL-13 increases ZEB1 expression and induces EMT markers changes in CRC cells, but the mechanisms remained largely unknown. It has been reported that IL-13 can activate several signaling pathways [34]. To clarify the activated signaling cascades by IL-13, HT29 and SW480 cells were stimulated with different doses of IL-13 for 1 h or with 100 ng/mL IL-13 for different times points. The immunoblotting experiments showed that IL-13 dramatically stimulated the phosphorylation of STAT6 and AKT, but not STAT3 or MAPK, while MAPK pathway was endogenously activated but not affected by IL-13 (Figure 3A, Figure S1A and S1B). Consistent with previous reports [35], almost all of phosphorylated STAT6 could translocate to the nucleus (Figure S1C and S1D). To determine which of the two pathways was engaged in EMT-related effects, the CRC cells were treated with JAK inhibitor JAKi1 (10 uM) or PI3K inhibitor LY294002 (0.1 uM) 1 h before IL-13 treatment (Figure 3B). We observed that the blockade of STAT6 phosphorylation by the pharmacological inhibitor JAKi1 significantly reversed IL-13-induced up-regulation of ZEB1 and Vimentin and down-regulation of E-cadherin, whereas inhibition of AKT activation did not influence the alteration of the EMT-related markers (Figure 3C and Figure S2), suggesting that IL-13-induced EMT changes are mediated by canonical JAK/STAT6 activation but not by the alternative pathway.

### STAT6 binds directly to the *ZEB1* gene promoter to activate its transcription in response to IL-13 stimulation

**Figure 4:**
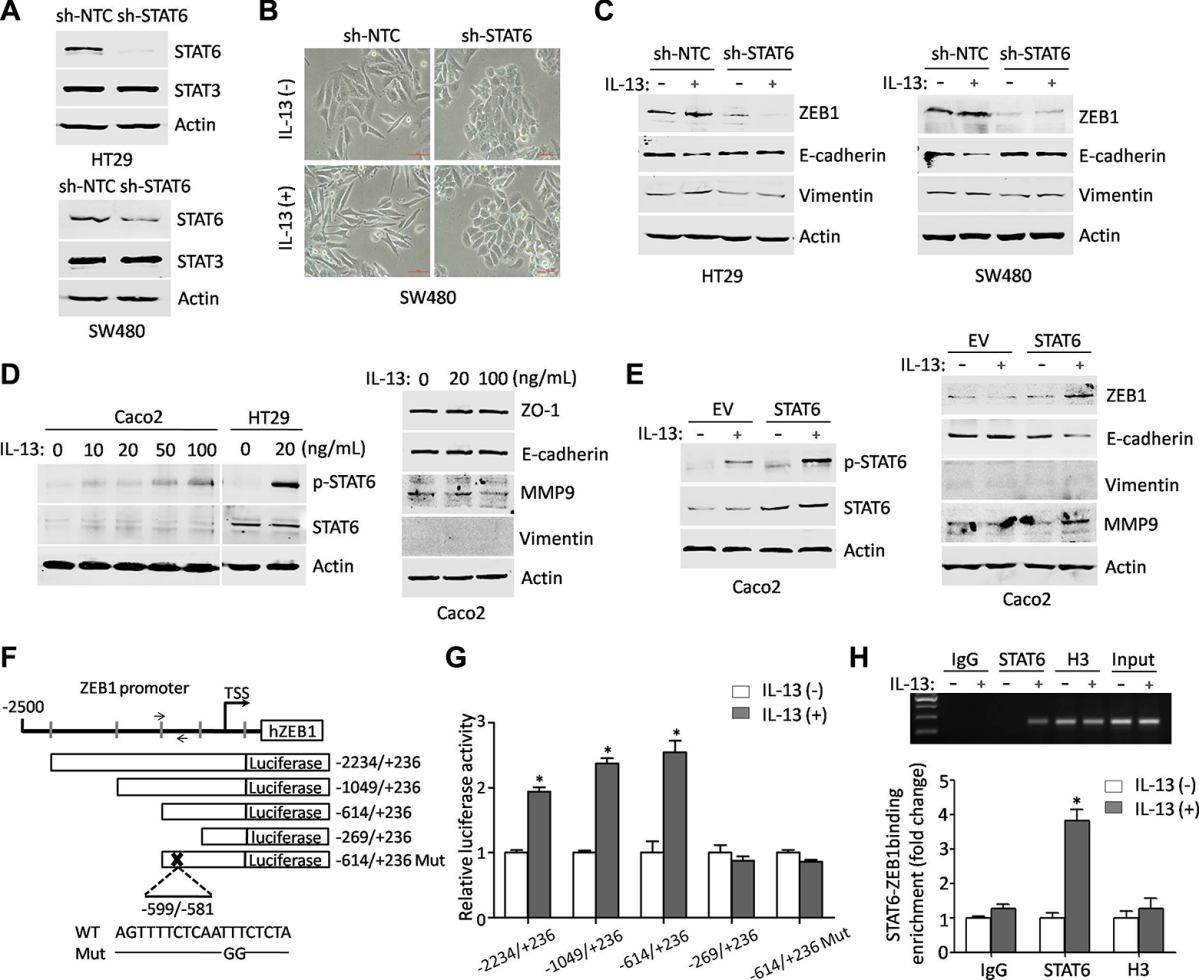
ZEB1 is transcriptionally regulated by STAT6 in response to IL-13 stimulation (**A**) Immunoblot analysis for specific knockdown of STAT6 in HT29 and SW480 cells. (**B**) Representative phase-contrast images of the maintained epithelial appearance in STAT6 knockdown SW480 cells treated with IL-13 for 72 h. Scale bar = 50 μm. (**C**) Immunoblot analysis for EMT markers in sh-NTC or sh-STAT6 HT29 and SW480 cells treated with or without 100 ng/mL IL-13 for 72 h. (**D**) (Left) Level of p-STAT6 was detected by western blots in Caco2 cells stimulated by gradient concentrations of IL-13 for 1 h. The level of p-STAT6 in HT29 cells exposed to 20 ng/mL IL-13 for 1 h was used as a positive control. (Right) Immunoblot analysis for EMT markers from 20 ng/mL or 100 ng/mL IL-13-treated Caco2 cells and control cells. (**E**) (Left) Western blots of the p-STAT6 and STAT6 expression in Caco2 cells transfected with STAT6 expression vector or empty vector and incubated with IL-13 (100 ng/mL) for 1 h afterwards. (Right) Immunoblot analysis for ZEB1, E-cadherin, Vimentin and MMP9 in control or STAT6-overexpression Caco2 cells treated with or without IL-13 (100 ng/mL) for 72 h. (**F**) Schematic presentation of *ZEB1* promoter with 5 potential SIEs and the primer pair used in ChIP-PCR or ChIP-qPCR. The reporter construct ZEB1-Luc and its truncated and mutated derivatives are also shown. (**G**) The relative luciferase activity of deletion mutants and SIE mutant of ZEB1-luc in HT29 cells treated by IL-13 (100 ng/mL) for 6 h. *P < 0.05. (**H**) ChIP assays were performed on HT29 cells exposed to IL- 13 (100 ng/mL) for 3 h with the indicated antibodies; RT-PCR and real-time PCR were applied to analyze the purified DNAs or soluble chromatin using specific primer pair for the *ZEB1* promoter.

To further confirm that STAT6 activation was required for IL-13-mediated EMT-related phenotypes, we sought to selectively suppress STAT6 expression using STAT6 shRNA (Figure 4A). HT29 and SW480 cells were transduced with lentiviral vectors carrying scrambled shRNA or specific shRNA against STAT6. The two stable shRNA pools expressing scrambled shRNA (sh-NTC) and STAT6 shRNA (sh-STAT6) were cultured in serum-free RPMI 1640 medium in the presence or absence of IL-13 for 72 h. As shown in Figure 4B, STAT6-knockdown SW480 cells demonstrated a more epithelial phenotype in the presence of IL-13, which was similar to that displayed by SW480 cells in the absence of IL-13. In contrast, SW480 cells treated with sh-NTC displayed spindle-shape morphology after IL-13 stimulation, which is similar to that of control (without shRNA treatment) SW480 cells after IL-13 stimulation (Figure 1A). More importantly, shRNA-mediated STAT6 knockdown also had a destructive effect on IL-13-induced ZEB1 expression and EMT changes in CRC cells (Figure 4C). On the contrary, IL-13 could not activate STAT6 phosphorylation or induce an EMT phenotype in Caco2 cells (Figure 4D), a STAT6^null^ CRC cell line reported in previous study [36], While ectopic expression of STAT6 obviously increased ZEB1 expression and induced EMT phenotypes in Caco2 cells exposed to IL-13 (Figure 4E). The promoter sequence of the *ZEB1* gene was analyzed and in which 5 potential STAT-inducible elements (SIEs) were predicted (Figure 4F). Serial deletion constructs of the *ZEB1* gene promoter were generated and examined by luciferase reporter assays to identify the transcriptional regulatory region responsive to IL-13/STAT6 signaling. It was shown that the *ZEB1* promoter without the region between −614 and −269 lost the ability to respond to IL-13 stimulation. This region contained one predicted SIE. Mutation in this SIE (from AATTTC to AATGGC) significantly abolished luciferase activity induced by IL-13 (Figure 4G). ChIP-PCR and ChIP-qPCR analysis further demonstrated that STAT6 directly bound to the *ZEB1* promoter region around −600 bp upstream of the transcription start site upon IL-13 exposure (Figure 4H). The evidence suggests that IL-13-activated STAT6 promotes transcription of the *ZEB1* gene by direct binding to the promoter.

### IL-13 promotes stem-like phenotypes and the migration and invasion of CRC cells through the STAT6 signaling

**Figure 5:**
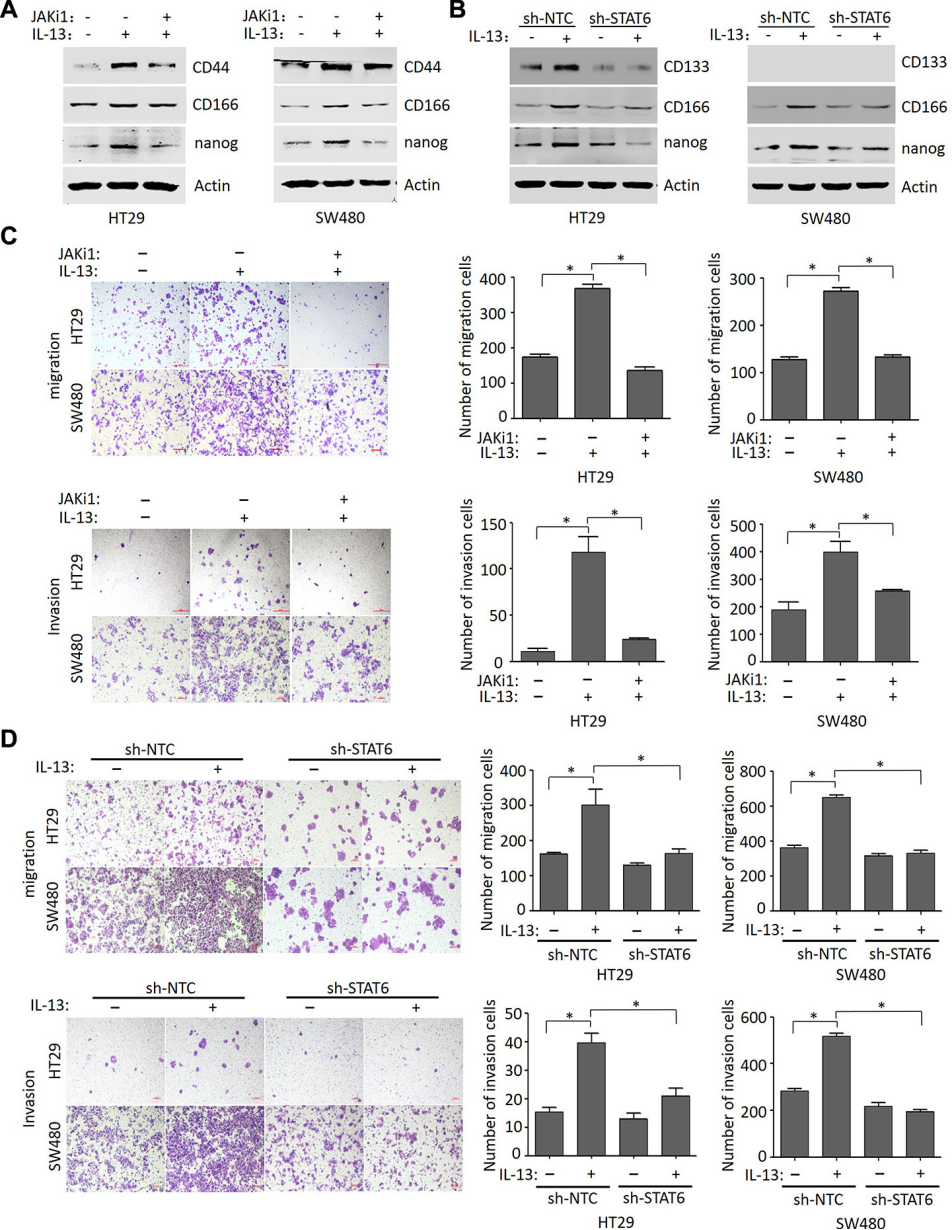
IL-13 promotes stem-like phenotypes and the migration and invasion of CRC cells through the STAT6 signaling (**A**) Immunoblot analysis for CD44, CD166 and nanog in HT29 and SW480 cells pretreated for 1 h with 10 μM JAKi1 and exposed to 100 ng/mL IL-13 for additional 72 h. (**B**) Expression of CD133, CD166 and nanog proteins in sh-NTC or sh-STAT6 HT29 and SW480 cells treated with or without 100 ng/mL IL-13 for 72 h. (**C**) Representative images of HT29 and SW480 cells induced by 100 ng/mL IL-13 or pretreated (1 h) with JAKi1 (10 uM) to move or invade through uncoated or matrigel-coated transwell membranes (quantification in right panels). Migration was analyzed at 48 h, and invasion at 72 h. Scale bar = 100 μm. Error bars represent SD. *P < 0.05. (**D**) The effect of STAT6 knockdown on the migration and invasion of HT29 and SW480 cells treated with or without 100 ng/mL IL-13 (quantification in right panels). Migration was analyzed at 48 h, and invasion at 72 h. Scale bar = 100 μm. Error bars represent SD. *P < 0.05.

Numerous findings have shown that EMT program endows cells with stem-like properties and enables cancer cell dissemination and metastasis [37]. To further investigate whether IL-13 promotes self-renewal of CRC cells, we evaluated the expression of several stemness makers in HT29 and SW480 cells treated with or without IL-13 in serum-free medium. We found that the expression of nanog, CD166 and CD44 were significantly up-regulated in IL-13-treated cells and reversed by inhibitor JAKi1 (10 uM) (Figure 5A). Likewise, silencing STAT6 in HT29 and SW480 cells also reversed IL-13-induced elevation of stem cell markers (nanog, CD166 and CD133) (Figure 5B). These results indicated that IL-13 induces cancer stem cells (CSCs) through STAT6 pathway. Next, we aimed to study the mechanism of IL-13 enhanced CRC cells migration and invasion. Transwell assays revealed that the IL-13-enhanced migratory and invasive abilities were obviously abrogated by JAK/STAT6 signaling inhibitor JAKi1 (10 uM) in HT29 and SW480 cells (Figure 5C). Additionally, suppression of STAT6 levels also counteracted IL-13-induced cell migration and invasion in HT29 and SW480 cells (Figure 5D). In converse, IL-13 promoted migration and invasion of STAT6-overexpressed Caco2 cells compared with the wildtype (Figure S3). Thus, IL-13 plays an important role in promoting aggressive behavior in CRC cells through the STAT6 signaling.

### IL-13Rα1/STAT6/ZEB1 signaling is important in EMT triggered by IL-13

**Figure 6:**
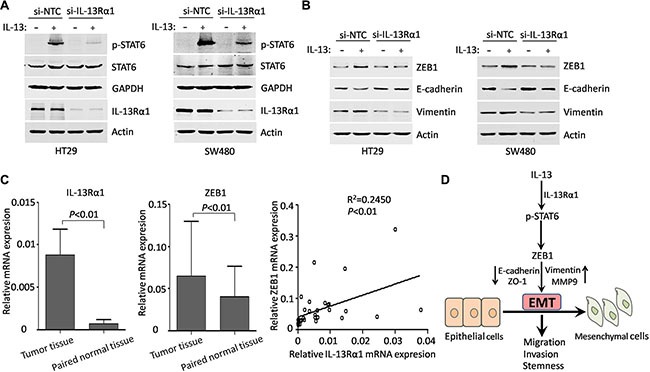
IL-13Rα1/STAT6/ZEB1 signaling is important in EMT triggered by IL-13 (**A**) Western blots of the effect of si-NTC, si-IL-13Rα1 on the expression of IL-13Rα1, STAT6 and p-STAT6 in HT29 and SW480 cells when treated with or without 100 ng/mL IL-13 for 1 h. (**B**) Western blots of ZEB1, Vimentin and E-cadherin from HT29 and SW480 cells transfected with control or IL-13Rα1 siRNA and incubated with IL-13 for additional 72 h. (**C**) Left panels: qRT-PCR detection of mRNA expression of IL-13Rα1 and ZEB1 in CRC tissues and adjacent non-cancerous tissues. Error bars represent SD. Right panel: linear regression between mRNA expression of IL-13Rα1 and ZEB1 in CRC tissues. (**D**) Simplified overview of IL-13/IL-13Rα1/STAT6/ZEB1 signaling that regulate EMT and aggressiveness in CRC.

It has been reported that IL-13 bind to IL-13Rα1 chains to initiate signal transduction through JAK/STAT6 pathways, while IL-13 signaling through IL-13Rα2 involves PI3K and ERK/AP1 pathways [29, 38]. Thus, we next determined the role of IL-13Rα1 in the IL-13 induced EMT. When IL-13Rα1-siRNA was successfully transferred into HT29 and SW480 cells, the phosphorylation of STAT6 stimulated by IL-13 was inhibited (Figure 6A). More importantly, siRNA-mediated IL-13Rα1 knockdown had a destructive effect on the EMT process in both CRC cell lines similar to that of STAT6 silencing (Figure 6B). To explore the expression of IL-13Rα1 and ZEB1 for CRC patients, real-time PCR assays were performed. This method indentified an increased mRNA expression of IL-13Rα1 and ZEB1 in 33 CRC samples compared with paired normal samples. Notably, increased IL-13Rα1 expression is positively correlated with ZEB1 expression in CRC samples (Figure 6C). These data suggest that IL-13/IL-13Rα1/STAT6/ZEB1 signaling is important for EMT process and CRC progression.

## DISCUSSION

Recent advances in the molecular cancer biology show that multiple molecules, including cytokines and their receptors, may mediate abnormal cellular signals through multi-pathways resulting in the aggressive biological behavior of cancer. IL-13 is a cytokine of increasing interest to gastroenterologist due to its developing role in inflammation and fibrosis in ulcerative colitis (UC) and Crohn's disease (CD) [8, 39]. Recurrent inflammation with ulceration and tissue restitution confers an increased risk of CRC in both UC and CD. IL-13 induced STAT6 phosphorylation is increased in UC, resulting in colon epithelial cell dysfunction [40, 41]. Thus, IL-13 can be referred to as a pro-oncogenic factor and IL-13 polymorphism (−1112C/T) has been identified as a higher risk of colon cancer occurrence [42, 43]. In the past few years, more and more attention has been drawn to the research on the connection of IL-13 and cancer metastasis. In pulmonary metastatic model of breast cancer, Th2 CD4^+^ T lymphocytes that produce IL-4 and IL-13, M2-type TAMs and IMCs are activated and in turn produce EGF, thus resulting in activation of a paracrine-mediated enhancement of malignant cell invasion and dissemination [44]. Thus far, the exact role and mechanism of IL-13 in colorectal cancer (CRC) metastasis remain unclear. Accumulating evidence has indicated a critical role of epithelial-mesenchymal phenotypic switch of tumor cells during cancer progression [45]. Here, we demonstrate that IL-13 is involved in the human CRC invasion and metastasis but not in proliferation, and that IL-13 is able to promote EMT changes and enhance aggressiveness of CRC cells through the previously unrevealed IL-13Rα1/STAT6/ZEB1 signaling (Figure 6D).

The IL-13 has been reported to be induced by TGF-β which is well known as a key mediator of EMT and plays a synergistic role with TGF-β in the pathogenesis of fistulae through promoting the expression of EMT-related molecules [32]. In CRC cells, kanai et al. demonstrated IL-13 inhibited cell-cell adhesion and down-regulated carcinoembryonic antigen (CEA) molecules, suggesting that IL-13 might be involved in the process of invasion or metastasis in CRC [46]. In this study, we find that IL-13 induces cellular morphological changes in human CRC cell lines HT29 and SW480 that are concomitant with up-regulated expression of mesenchymal marker Vimentin and down-regulated expression of epithelial marker E-cadherin, suggesting that IL-13-treated cells undergo EMT-related changes. In addition, IL-13 significantly promotes the expression of EMT core regulator ZEB1, and ZEB1 knockdown reverses the IL-13-induced EMT, indicating that IL-13 acts through ZEB1 to induce EMT in CRC cells. Furthermore, IL-13-induced EMT cells display enhanced migratory and invasive capacity when compared with parental cells with an epithelial phenotype.

In our model, we find that IL-13 induces STAT6 and PI3K activation. However, only STAT6 signaling inhibitor JAKi1 blocks IL-13 effects on HT29 and SW480 cells. It indicates that IL-13 may act through STAT6 activation to induce EMT and aggressiveness in CRC cells. The dependence of STAT6 is further confirmed by knocking down STAT6 in HT29 and SW480 cells, which display a reverse of IL-13-mediated EMT changes. Similarly, IL-13 can't activate STAT6 phosphorylation or induce an EMT phenotype in Caco2 cells (STAT6 deficient). Previous study has revealed that HT29 cells with active STAT6 signaling harbor a microenvironment favoring Th2 cytokines and promoting expression of genes related to pro-growth, pro-metastasis and anti-apoptosis, but Caco2 cells carrying defective STAT6 signaling exhibit spontaneous expression of anti-metastatic genes [47]. However, ectopic expression of STAT6 in Caco2 cells promotes IL-13-induced EMT. Increased p-STAT6 has also been shown in ulcerative colitis (UC) patients and is involved in IL-13-induced colon epithelia dysfunction [41]. Besides, constitutive activation of STAT6 is found in a variety of malignancies, including mediastinal primary large B-cell lymphoma [48], Hodgkin's lymphoma [49], prostate cancer [50] and cutaneous T-cell lymphoma [51]. Conversely, lacking STAT6 in mouse model enhances tumor immunity to both primary and metastatic mammary carcinomas [52]. Down-regulation of STAT6 in a prostate cancer cell line results in reduced cell viability, induced apoptosis and impaired migration [53]. Studies have identified transcription factors, various kinases, kinase inhibitors, other enzymes, cytokines, cell surface receptors, and other genes under the regulatory control of STAT6 [35]. To our knowledge, we have, for the first time, characterized the involvement of ZEB1 in the transcriptional activity of IL-13-activated STAT6. Using ChIP and dual promoter gene assays (truncation and mutation), we map a highly active promoter region located between −614/−269 of *ZEB1*. Therefore, STAT6 activity is required for tumor cells aggressiveness. However, we don't find IL-13/STAT6 signaling promote cell growth in our model. Similarly, IL-13 can't promote the growth of lung vascular SMCs, whereas it significantly promotes the growth of airway SMCs. Interestingly, IL-13 could induce phosphorylation of STAT6 in both airway and vascular SMCs [54]. The discrepancy may depend on target genes in the context of different malignancy.

Furthermore, we also demonstrate IL-13 can promote stem cell properties in the current study. Many studies have provided strong evidence of pro-CSC-forming role of EMT inducers [55]. Both CD44 and CD133 are known as putative markers for colorectal CSCs and CD133^+^ CD44^+^ tumor cell populations are responsible for liver metastasis [56]. Interestingly, CD44, CD166 and nanog have also been implicated to promote EMT [57–59]. In previous studies, IL-13 has been reported to increase CD44 and CD44 isoform expression in a murine model of asthma and in colon epithelial cells, respectively [60, 61]. In the present study, we further find that IL-13 induce up-regulation of CD44, CD166 and nanog which is reversed by inhibitor JAKi1. Similarly, knockdown of STAT6 can relieve the elevation of CD133, CD166 and nanog resulting from the stimulation of IL-13 in CRC cells. Taken together, IL-13 promotes stem cell markers expression through STAT6 signaling.

The biological effects of IL-13 are thought to be mediated by a shared receptor composed of the IL-13Rα1 and IL-4Rα chains [6], and a so-called decoy receptor attributed to the high affinity of IL-13Rα2 protein [62]. Multiple mechanisms seem to be operational in IL-13-induced cancer invasion and metastasis. IL-13 has been shown to act as an autocrine growth factor in pancreatic cancer that promotes lymph node metastasis [63] and is a major regulator of M2 macrophages to suppress immune surveillance in metastasis [64]. Interestingly, IL-13Rα2 but not IL-13Rα1 was reported to be involved in pancreatic and breast cancer metastasis [28, 65]. In CRC, IL-13Rα2 was also reported to highly express in highly metastatic cells and promote invasion and metastasis [27]. Surprisingly, here we detect low expression of IL-13Rα2 mRNA and protein expression in most CRC cell lines. Conversely, IL-13Rα1 is highly expressed in most CRC cell lines (Figure S4). In CRC clinical samples, we also find IL-13Rα1 expression level but not IL-13Rα2 is higher in CRC tissues when compared with adjacent non-tumor tissues at the mRNA levels. Furthermore, silencing IL-13Rα1 inhibits IL-13-mediated EMT changes in human CRC cell lines HT29 and SW480 through blocking STAT6 activation. Consistently, previous studies also reported that IL-13Rα2 mRNA did not be detected by RT-PCR in HT29/B6 colon cancer cells and IL-13/IL-13Rα1 pathway was shown to play a central role in the regulation of intestinal epithelial architecture and function [66]. In addition, IL-13 does not activate Erk1/2 signaling in our model, which is consistent with the previous reports that IL-13 activated Erk1/2-MAPK pathway through IL-13Rα2 [28, 29].

Our findings demonstrate that IL-13 promotes EMT and cancer stem cell development in CRC cells, which contribute to CRC malignancy. Mechanistic studies revealed that transactivation of ZEB1 was induced by IL-13 through activating the phosphorylation of STAT6. Furthermore, we highlight the role of IL-13Rα1/STAT6/ZEB1 signaling in the IL-13-induced EMT phenotypes and aggressiveness of CRC. However, Whether EMT plays a crucial role in cancer metastasis has been heavily debated [67–69], largely due to the difficulty to observe the occurrence of EMT *in vivo* and track the fate of cells undergoing EMT in clinical settings as well as the diversity of the EMT program that can elude detection using a single EMT marker in animal models [70]. EMT is also considered as a transient process and tumor cells need to undergo the reverse process, mesenchymal-to-epithelial transition (MET), to successfully colonize a distant organ [71]. Besides, the non-physiological overexpression or complete loss of EMT-regulators, such as Twist1 or Snail1, may induce expression profiles and subsequently cell phenotypes that do not exist under physiological conditions [72]. In our work, we didn't perform any animal experiments. We hope to further verify our results in better models. Recently, compelling evidence has reported that tumor microenvironment dominates EMT development and impacts cancer metastasis [73]. Studies have shown that IL-13 mediates extracellular matrix proteins (Fibronectin and collagen I) production by fibroblasts via STAT6 pathway [74, 75], and aggressive colon tumors with collagen-rich stroma present mesenchymal features and stronger capability for invasion [76]. Further, M2-like polarized tumor-associated macrophages (TAMs), which can be induced by IL-13, promote EMT and cancer metastasis [77, 78]. Based on these, our future studies may be focus on the exact relationships between IL-13 and various types of cells (such as fibroblasts and macrophages) in the microenvironment of CRC. Besides, IL-13 has been implicated in inflammation and remodeling by inducing chemokines (CCL-3, CCL-4, CCL-5 and CXCL-1) [79], and IL-13 is a direct inducer of both CCL11 and CCL24 in eosinophilic esophagitis [80]. Further characterization of the functional and mechanistic relationship between IL-13 and other inflammatory factors in cancer progression remains to be explored.

In conclusion, our data reveal that the aggressive properties of IL-13 in CRC were mediated through STAT6-denpendent pathway, and STAT6 directly binds to the promoter of *ZEB1* and transcriptional activates its expression and induces EMT process (Figure 6D). IL-13 may be considered as a novel therapeutic target for CRC in the clinic.

## MATERIALS AND METHODS

### Cells and cell culture

The human CRC cell lines HT29 and SW480 were purchased from the American Type Culture Collection (ATCC, Manassas, VA, USA) and maintained in RPMI 1640 medium supplemented with 10% heat-inactivated fetal bovine serum (FBS, Hyclone, Tauranga, New Zealand). The Caco2 cell and the 293T/17 cell were purchased from the cell bank at the Chinese Academy of Sciences (Shanghai, China) and grown in Dulbecco's modified Eagle's medium (DMEM) with 10% FBS. All cells were authenticated by STR profiling before distribution and never passaged longer than 6 months. All cells were cultured in a humidified incubator with 37°C and 5% CO_2_.

### Antibodies and reagents

The primary antibodies used in the immunoblotting against E-cadherin, Twist1, STAT6, STAT3, GAPDH and Actin were from Santa Cruz Biotechnology (Santa Cruz, CA, USA). The N-cadherin, Vimentin, ZO-1, Snail1, Slug, ZEB1, MMP9, p-STAT6 (Tyr641), p-STAT3 (Tyr705), AKT, p-AKT, Erk1/2, p-Erk1/2, nanog and CD44 antibodies were from Cell signaling Technology (Beverly, MA, USA). Fibronectin antibody was from Millipore (Billerica, MA, USA). CD166 and histone H3 antibodies were from Abcam (Cambridge, UK). CD133 antibody was from MiltenyiBiotec (GmbH, Bergisch Gladbach, Germany). IL-13Rα1 and IL-13Rα2 antibody were from Sangon Biotech (Shanghai, China). The primary antibody used in the immunofluorescence against E-cadherin and Vimentin were from Cell signaling Technology (Beverly, MA, USA) and DAKO (Carpinteria, CA, USA), respectively. The pharmacological reagents used in this study are the following: recombinant human IL-13 (Peprotech, Rocky Hill, NJ, USA); JAK inhibitor 1 (JAKi1) and LY294002 (Calbiochem, San Diego, CA, USA).

### Cell morphological observation

The cells treated or untreated with IL-13 (100 ng/mL) were observed and photographed under inverted microscope (Eclipse Ti, Nikon, Kyoto, Japan). To further observe the cellular microvilli or pseudopodium, scanning electron microscope was used. Briefly, the cells were washed with PBS (phosphate-buffered saline) and fixed in 2.5% glutaraldehyde 0.1 M PBS (pH 7.4) overnight. Washed in PBS and fixed in 1% osmic acid in 0.1 M PBS (pH 7.4) at 4°C for 1 h. Washed again in PBS, the cells were progressively dehydrated in ethanol and dried in acetonitrile solution. The cells were sprayed with gold and then were photographed under the scanning electron microscope (Hitachi S-3000N, Japan).

### RNA extraction and quantitative real-time PCR (qRT-PCR)

Total RNA was isolated using TRIzol reagent (Invitrogen, Carlsbad, CA, USA) and reverse transcription was carried out using PrimeScript^™^ RT reagent Kit (TaKaRa, Dalian, China). The quantitative real-time PCR assays were performed to analyze mRNA levels by using an SYBR Green/ROX PCR Master Mix (TaKaRa) on an ABI Prism 7900 Sequence Detection System (Applied Biosystem, Foster City, CA, USA). All Primers used for qRT-PCR analysis were synthesized by Sunny Biotech, Shanghai, China. The sequences are listed in Table S1. Samples were normalized to GAPDH and ΔΔCt methods were used to calculate fold expression changes of mRNA.

### Immunoblotting

The cell proteins were extracted using an extraction kit (Beyotime, Haimen, Jiangsu, China) according to the recommended protocol. The protein concentrations were determined using the Bradford method with a Bio-Rad protein Assay kit (Bio-Rad, Hercules, CA, USA). The protein samples (50 μg) were heated to 100°C for 5 min in 2× loading buffer and separated by 8% to 12% SDS-PAGE, followed by transfer to Nitrocellulose membranes (Bio-Rad) with a Bio-Rad transfer unit (Bio-Rad). The membranes were blocked with TBST buffer (Tris buffered saline plus 0.05% Tween-20) containing 5% w/v nonfat milk, incubated overnight with primary antibody at 4°C, and then incubated with a specific secondary antibody conjugated with either LI-COR IR-680 or LI-COR IR-780 antibody for 2 h at room temperature (RT). Protein bands were visualized by the Odyssey Infrared Imaging System (LI-COR Biosciences, Lincoln, NE, USA).

### Immunofluoresence

Cells were cultured in chambers and treated with recombinant IL-13 (100 ng/mL) for 72 h. At the end of incubation, cells were fixed with 4% paraformaldehyde for 20 minutes at RT and permeabilized with 0.03% Triton in PBS. Next, slides were washed with PBS, blocked with 5% donkey serum for 1 h at RT and incubated overnight at 4°C with rabbit anti-E-cadherin and mouse anti-Vimentin in blocking buffer. After washing with PBS, chamber slides were incubated with secondary antibodies (donkey anti-mouse-IgG-Alexa Fluor 488 and donkey anti-rabbit-IgG-Alexa Fluor 594) (Invitrogen). Slides were then counterstained with DAPI (Sigma, St Louis, MO, USA) and mounted with Prolong gold antifade Reagent (Invitrogen). The fluorescent images were captured using a confocal microscope (BX61W1-FV1000, Olympus, Tokyo, Japan). The images are representative of three independent experiments.

### Wound healing assay

Cells were cultured in 6-well plates containing RPMI 1640 with 10% FBS until they become 90% confluent with complete medium. The media was then removed and two wounds per well were made by scraping with pipette tips. The plates were washed twice with PBS to remove cellular debris and added with serum-free RPMI 1640 with or without 100 ng/mL IL-13. Pictures from the same area of the wound were taken under the inverted microscope (Nikon) at different time points after scraping. For each wound, the distance of the gap was the average of four fields. The measurement for six wounds per treatment were collected and analyzed statistically.

### Cell proliferation assay

Cells were seeded at a density of 2000 cells per well in 96-well plates and incubated. An aliquot of 10 μl of CCK8 (Boster, Wuhan, China) was added to the wells and incubated for 3 h. The absorbance was measured at 570 nm to calculate the numbers of viable cells in each well. Each measurement was performed in triplicate and the experiments were repeated thrice.

### Transwell migration and matrigel invasion assays

The 8-μm transwell 24-well chambers (Costar, Cambridge, MA, USA) were used for *in vitro* cell migration and invasion assays. For the migration assay, cells were resuspended in serum-free RPMI 1640 with or without 100 ng/mL IL-13 and seeded in the upper compartment of the chamber (200 μL/well), whereas 600 μL complete RPMI 1640 with 10% FBS was prepared in the lower chamber. The invasion assay was performed in the transwell chambers coated with Matrigel (BD Biosciences, San Jose, CA, USA), and then cells were seeded as described before. For the migration assay, HT29 (8 × 10^4^ cells/well) and SW480 (4 × 10^4^ cells/well) were used. For the invasion assay, HT29 (2 × 10^5^ cells/well) and SW480 (8 × 10^4^ cells/well) were used. Notably, JAKi1 was prepared with serum-free RPMI 1640 medium and then used to suspend the cells. Cells on the upper side of the filter were removed by cotton swab. Those on the lower surface were fixed with 4% paraformaldehyde and stained with 0.1% crystal violet after 48 h for the migration assay or 72 h for the invasion assay. Cells that migrated or invaded were counted from five randomly selected fields under an inverted microscope (Nikon) and the experiments were repeated thrice.

### Gelatin zymography

HT29 and SW480 cells were incubated overnight in serum-free medium and the proteins in the conditioned medium were concentrated with Amicon Ultra-4 (Millipore). Proteins were loaded without boiling onto 10% polyacrylamide gel containing gelatin (Sigma) at 1 mg/mL. The gels were shaken in a renaturing solution of 2.5% Triton X-100 for 1 h, and then developed 42 h at 37°C in incubation buffer (50 mM Tris-HCl, pH 7.6, 50 mM NaCl, 5 mM CaCl_2_ and 1 μM ZnCl_2_). The gels were stained by 0.05% Coomassie Brilliant Blue R250 for 2 h and destained to the desired degree. Gelatinolytic activity was visualized as bright areas in the gel, and images were captured using the Odyssey Infrared Imaging System.

### Stable knockdown of STAT6

Human STAT6 shRNA in pLKO.1 vector was purchased from Open Biosystems. As shRNA control vectors, we used a scrambled shRNA and an empty pLKO.1 vector obtained from Addgene. Lentiviruses expressing STAT6 shRNA were produced in 293T/17 cells packaged by pMD2G and psPAX2 with LipoD293^™^ DNA *in vitro* transfection reagent (SignaGen Laboratories, Gaithersburg, MD, USA) according to the manufacturer's instructions. SW480 and HT29 cells were infected with scrambled or STAT6 shRNA lentiviral particles containing 12 μg/mL polybrene (Sigma). After incubation 72 h, Stable infected cells were established via selection with 10 μg/mL puromycin (Sigma).

### Stable overexpression of STAT6

The cDNA of full-length human STAT6 was cloned into the modified lentiviral pMIRNA1-IRES-GFP expression vector, a gift from Dr. Rongpan Bai (Zhejiang University, Hangzhou, China), and verified by bidirectional sequencing. Lentiviruses were produced in 293T/17 cells with pCMVΔ8.9 and pVSVG with LipoD293^™^ DNA *in vitro* transfection reagent mentioned above. Caco2 cells infected with virus particles were subsequently fluorescence activated cell sorting analyzed for green fluorescent protein to obtain stable cell lines.

### Interfering RNA (RNAi)

siRNAs were as follows: ZEB1 siRNA targeted the sequence 5′-GGCGGTAGATGGTAATGTA-3′; IL-13Rα1 siRNA targeted the sequence 5′-CCGGAAAC TCGTCGTTCAA-3′; 5′-TTCTCCGAACGTGTCACGT-3′ was control siRNA. All the siRNA oligonucleotides were designed and synthesized by Genepharma (Genepharma Co.Ltd, Shanghai, China). Cell transfection was performed using PowerFect^™^ *in vitro* siRNA transfection Reagent (SignaGen). For siRNA knockdown and transient transfection experiments, RNA or protein extraction was performed 48 h post-transfection.

### Luciferase reporter assay

Promoter fragments of *ZEB1* (−2234/+236, −1049/+236, −614/+236 and −269/+236) were subcloned into the MluI/XhoI sites of the pGL3 vector (Promega, Madison, WI, USA). The construct of mutant ZEB1 (−614/+236 MUT), which carried a replacement of two nucleotides within the binding sites, was generated through site-directed mutagenesis (Stratagene). HT29 cells were transiently transfected with reporter constructs. The pRL-SV40 vector was co-transfected in each experiment as an internal control. At 48 h post-transfection, cells were incubated with 100 ng/mL IL-13 for 6 h and the luciferase activities were measured using the Dual-Luciferase Reporter Assay Kit (Promega). All of the experiments were carried out in triplicate.

### Chromatin immunoprecipitation (ChIP)

HT29 cells were serum-starved overnight and treated with 100 ng/mL IL-13 for 3 h. Cells was cross-linked with 1% formaldehyde, lysed and sonicated. Sheared chromatin was subjected to immunoprecipitation overnight at 4°C with anti-STAT6 or anti-histone H3, or with normal IgG (Santa Cruz). IgG was used as the negative control antibody, whereas anti-histone H3 and the chromatin extract without any antibody treatment were used as the positive controls. Chromatin-antibody complexes were isolated using Protein A/G PLUS-Agarose (Santa Cruz). The crosslinking was reversed and genomic DNA fragments were purified and analyzed by PCR or real-time PCR (qPCR) using the following primer pair for *ZEB1* promoter: 5′- CCGGTCACGTTTCAGTTT-3′ (forward) and 5′-TCCTGCTTCCCACCTCCT-3′ (reverse). All of the experiments were repeated at least three times.

### Clinical data

In this study, a total of 33 CRC tissues as well as their paired normal tissues were obtained in the affiliated hospital, Zhejiang University School of Medicine from 2001 to 2004. The patients were enrolled with informed consent. The study was approved by the Ethics Committee of Zhejiang University. The clinico-pathological characteristics of the clinical specimens are summarized in Table S2. Total RNA was isolated from the fresh specimens by using TRIzol reagent (Invitrogen) and reverse transcription was carried out using PrimeScript^™^ RT reagent Kit (TaKaRa). The cDNA of samples were stored at −80°C until use.

### Statistical analysis

The statistical analyses were conducted using SPSS Statistics software (Version.19.0; SPSS Inc., Chicago, IL, USA) Student's *t*-test was performed to compare paired data. The correlation between the levels of IL-13Rα1 and ZEB1 levels was determined using Pearson's correlation test. *P* < 0.05 was considered statistically significant.

## SUPPLEMENTARY MATERIALS FIGURES AND TABLES


