# Supplementary material for: IL-13/STAT6 signaling plays a critical role in the epithelial-mesenchymal transition of colorectal cancer cells
Source: Oncotarget. 2016 Aug 13;7(38):61183–98. doi: 10.18632/oncotarget.11282 (PMC5308644; doi:10.18632/oncotarget.11282)
Supplement: Supplementary file 1 [file oncotarget-07-61183-s001.pdf]

IL-13/STAT6 signaling plays a critical role in the epithelial-mesenchymal transition of colorectal cancer cells

Supplementary Materials

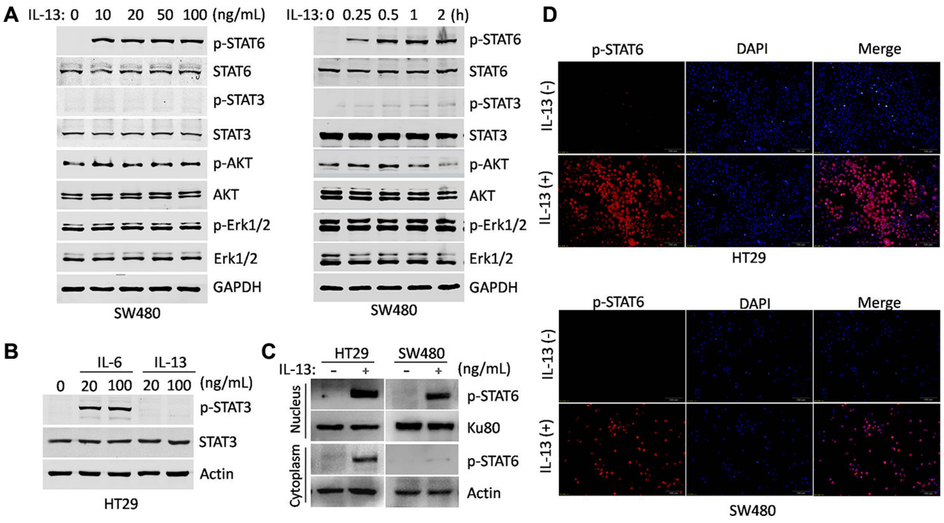

Supplementary Figure S1: The STAT6 and AKT signaling pathways were activated by IL-13 in CRC cells. (**A**) SW480 cells were treated with gradient concentrations of IL-13 for 1 h (Left) and 100 ng/mL IL-13 for different time points (Right). The levels of p-STAT6, p-STAT3, p-AKT and p-Erk1/2 were confirmed by western blot. STAT6, STAT3, AKT, Erk1/2 and GAPDH were used as sample loading controls. (**B**) Immunoblot analysis for p-STAT3 from HT29 cells treated with IL-13 or IL-6. STAT3 and Actin were used as sample loading controls. (**C**) Immunoblot analysis for p-STAT6 in Nucleus and cytoplasm from cells treated with IL-13. Ku80 and Actin were used as sample loading controls. (**D**) Immunofluorescent staining of p-STAT6 expression in HT29 and SW480 cells treated with IL-13 (100 ng/mL) for 1 h (nuclei stained with DAPI, 400×).

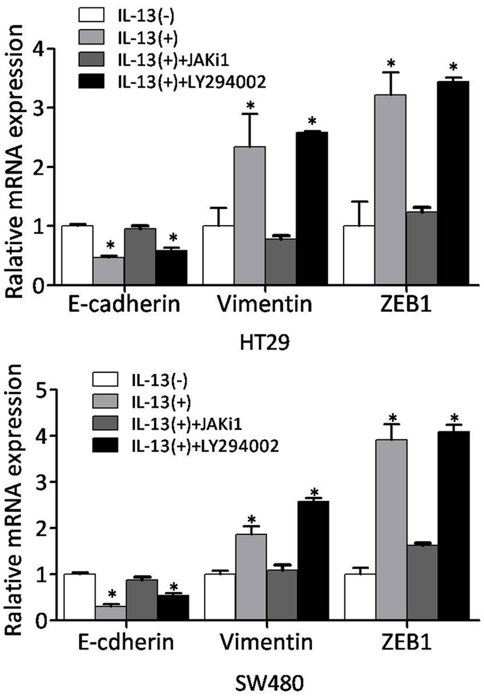

Supplementary Figure S2: IL-13-induced EMT changes are mediated by JAK/STAT6 activation. A. The mRNA levels of EMT molecular markers in CRC cells in HT29 and SW480 cells pretreated for 1 h with 10 μM JAKi1 or 0.1 μM LY294002 and exposed to 100 ng/mL IL-13 for additional 72 h. $*P < 0.05$.

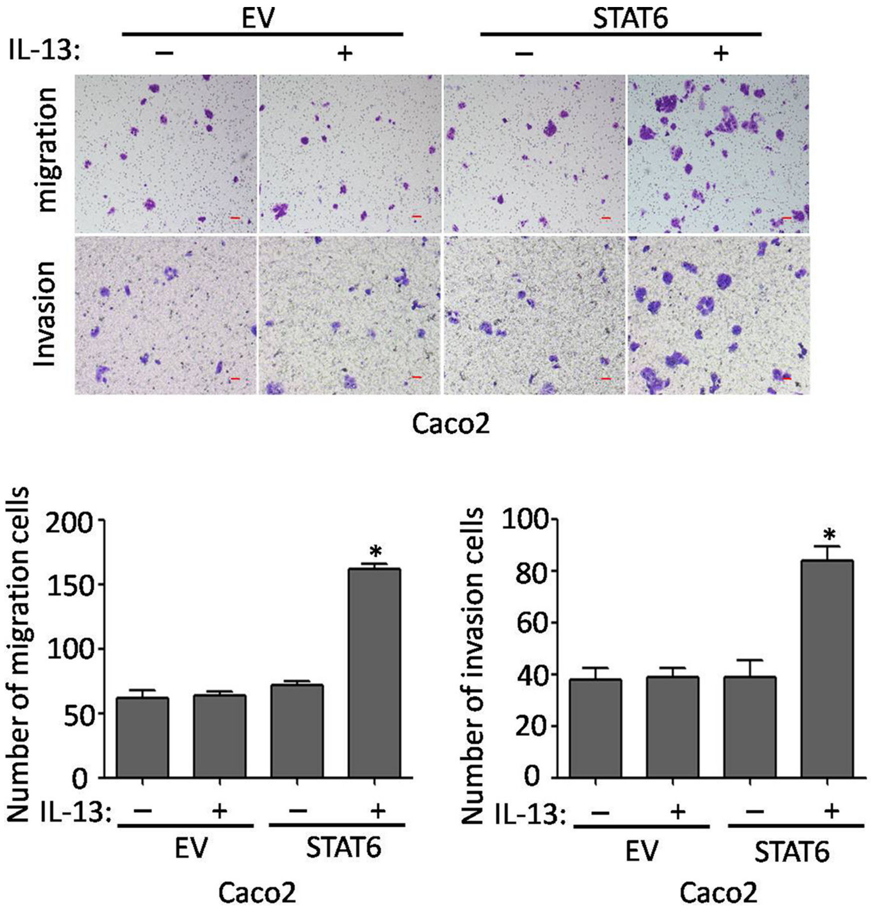

Supplementary Figure S3: IL-13 promotes the migration and invasion abilities in STAT6-overexpressed Caco2 cells. A. The effect of STAT6 overexpression on the migration and invasion of Caco2 cells treated with or without 100 ng/mL IL-13 (quantification in right panels). Migration was analyzed at 48 h, and invasion at 72 h. Scale bar = 100 μm. Error bars represent SD. **P* < 0.05.

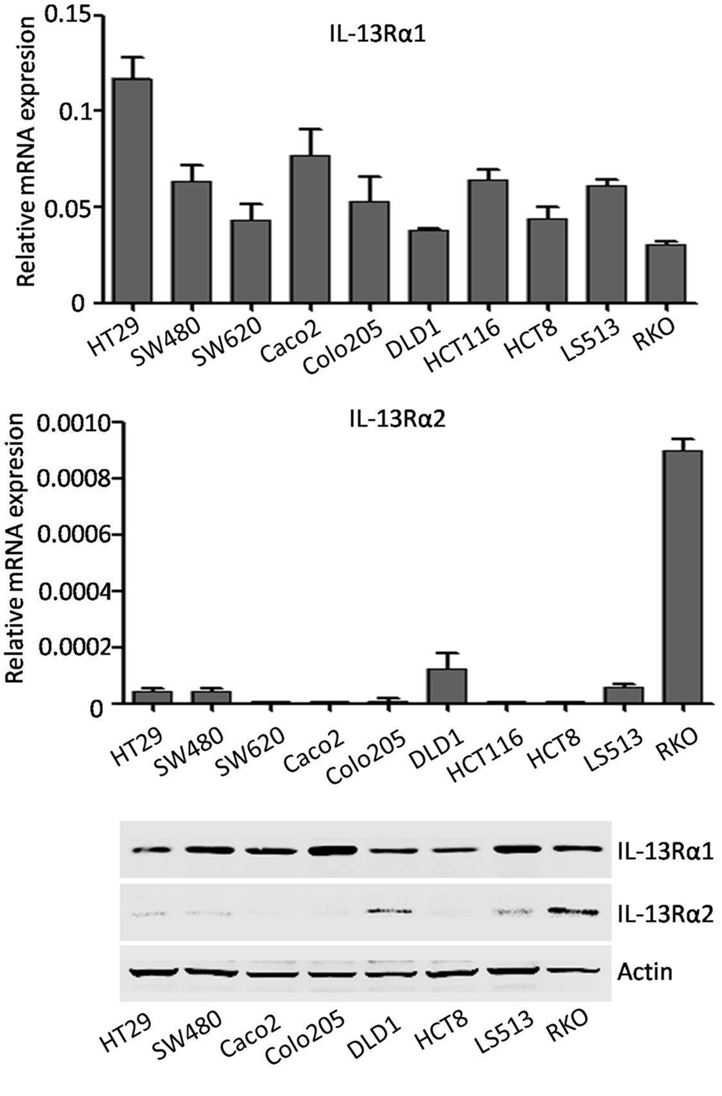

Supplementary Figure S4: The expression levels of IL-13Rα1 and IL-13Rα2 in colorectal cancer cells. A. Real-time PCR (upper panels) and western blot analysis (lower panels) of IL-13Rα1 and IL-13Rα2 expression in colorectal cancer cells.

Supplementary Table S1: Primer sequences for qRT-PCR analysis

Gene	Primer	Sequence	PCR products (bp)
IL-13Rα1	Sense	5′-GTGCCTTTAACTTCCCGTGT-3′	85
	Antisense	5′-CCCATTGCACATATAGGTCATC-3′	
IL-13Rα2	Sense	5′-ACCTGGCATAGGTGTACTTCT-3′	133
	Antisense	5′-CCAAATAGGGAAATCTGCATCCT-3′	
STAT6	Sense	5′-CCTCGTCACCAGTTGCTT-3′	214
	Antisense	5′-TCCAGTGCTTTCTGCTCC-3′	
E-cadherin	Sense	5′-ATCCAAAGCCTCAGGTCATA-3′	371
	Antisense	5′-CAGCAAGAGCAGCAGAAT-3′	
ZO-1	Sense	5′-TGGCCACAGCCCGAGGCATAT-3′	240
	Antisense	5′-GTAAGCGCAGCTCCACAGGC-3′	
Vimentin	Sense	5′-GAACTTTGCCGTTGAAGCTG-3′	143
	Antisense	5′-TCTCAATGTCAAGGGCCATC-3′	
Fibronectin	Sense	5′-AGTGCATCTGCACAGGCAACGG-3′	201
	Antisense	5′-TCAGCCACTGCATCCCCACAGA-3′	
N-cadherin	Sense	5′-AGCCAACCTTAACTGAGGAGT-3′	136
	Antisense	5′-GGCAAGTTGATTGGAGGGATG-3′	
Snail	Sense	5′-CTAGGCCCTGGCTGCTACAAG-3′	225
	Antisense	5′-AGCGGGGACATCCTGAGCA-3′	
Slug	Sense	5′-TGCGGCAAGGCGTTTTCCAGA-3′	239
	Antisense	5′-CAGTGTGCTACACAGCAGCCAGA-3′	
ZEB1	Sense	5′-GCACAACCAAGTGCAGAAGA -3′	190
	Antisense	5′-CATTTGCAGATTGAGGCTGA -3′	
Twist	Sense	5′-CTGCCCTCGGACAAGCTGAG-3′	199
	Antisense	5′-CTAGTGGGACGCGGACATGG-3′	
MMP-9	Sense	5′-GACCTCAAGTGGCACCACCA-3′	440
	Antisense	5′-GTGGTACTGCACCAGGGCAA-3′	
GAPDH	Sense	5′-ACCACAGTCCATGCCATCAC-3′	452
	Antisense	5′-TCCACCACCCTGTTGCTGTA-3′	

Supplementary Table S2: Clinico-pathologic parameters of colorectal cancer patients

Characteristics	Number(n = 33)
Age (years)	
≤ 60	19
> 60	14
Gender	
Male	15
female	18
Tumor location	
Colon	17
Rectum	14
Unknown	2
TNM Stage	
I+II	6
III	19
IV	4
Unkown	4
